# Precision over quantity: identifying trustworthy genomic risk stratification models

**DOI:** 10.31744/einstein_journal/2025CE1726

**Published:** 2025-07-11

**Authors:** Valbert Oliveira Costa, Pedro Robson Costa Passos

**Affiliations:** 1 Center of Research and Drug Development Universidade Federal do Ceará Fortaleza CE Brazil Center of Research and Drug Development, Universidade Federal do Ceará, Fortaleza, CE, Brazil.

Dear Editor,

The Brazilian Society of Medical Genetics and Genomics recently issued a statement against using polygenic risk scores for breast cancer risk stratification in Brazilian women.^1^ In fact, an ongoing surge could be observed in genomic signatures and prognostic models, with over 200 LASSO-based TCGA signatures appearing in *Scientific Reports* last year, based on a focused keyword-based search, several of questionable quality. Discerning reliable models from unreliable ones is crucial for clinicians in order to avoid poorly validated tools.

The first step is to assess how the model was built and whether it was properly validated. Currently, we distinguish two common validation types. The first involves splitting the patient dataset into proportions such as 80/20 or 70/30 for model training and testing. Unfortunately, this approach is less effective, as machine learning after a single random training-test split might yield unreliable results.^2^The second and more robust method involves validation using an entirely external dataset from independent studies. This approach is more reliable, as internal data splitting often fails to truly validate a model and cannot replace independent replication across different researchers, populations, and methodologies.^3^

The next step is the assessment of the area under the receiving operating curve (AUC), representing the integral of all points along the curve and capturing both sensitivity and specificity to provide a comprehensive measure of the overall performance of the model. In general, a model with an AUC <0.6 could be considered unreliable and acceptable models should display an AUC of at least 0.7.^4^Subsequently, evaluating the biological plausibility of the genes included in the model is essential. For instance, models in which a tumor suppressor expression predicts worse survival or an oncogene expression predicts better prognosis, could lack reproducibility. Models and signatures should (or should aim to) retain biological relevance and plausibility. Therefore, assessing whether the applied genes have been validated in the disease through methods beyond transcriptomics (*e.g.*, real-time PCR or immunohistochemistry) is also necessary.

Finally, most models and signatures are developed using publicly available datasets, such as those from TCGA or the Gene Expression Omnibus, and are typically produced and evaluated retrospectively. To ensure reproducibility and enable the integration of these models into clinical practice, model validation in prospective studies would be essential. This step is crucial, as prospective validation provides an important first assessment of the real-world machine learning model performances.^5^

**Figure 1:**
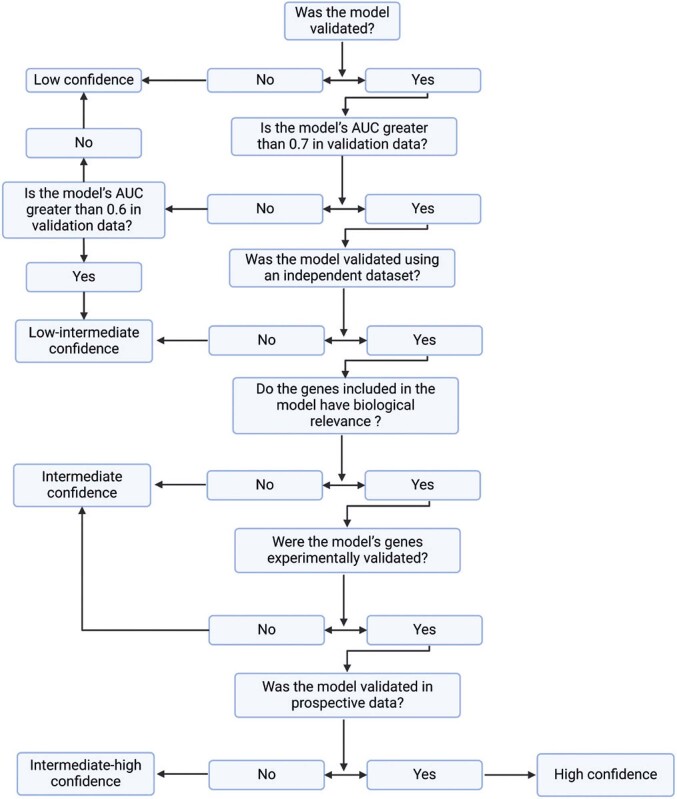
Algorithm for identifying robust models

These steps could potentially prove particularly valuable for clinicians, helping them avoid poorly designed studies, as this emerging field is underrepresented in the undergraduate curricula and its literature could be hard to navigate. Figure 1 presents a flowchart, summarizing the algorithm for identifying robust models.
